# Profound Activity of the Anti-cancer Drug Bortezomib against *Echinococcus multilocularis* Metacestodes Identifies the Proteasome as a Novel Drug Target for Cestodes

**DOI:** 10.1371/journal.pntd.0003352

**Published:** 2014-12-04

**Authors:** Britta Stadelmann, Denise Aeschbacher, Cristina Huber, Markus Spiliotis, Joachim Müller, Andrew Hemphill

**Affiliations:** Institute of Parasitology, University of Berne, Vetsuisse Faculty, Berne, Switzerland; University of Würzburg, Germany

## Abstract

A library of 426 FDA-approved drugs was screened for in vitro activity against *E. multilocularis* metacestodes employing the phosphoglucose isomerase (PGI) assay. Initial screening at 20 µM revealed that 7 drugs induced considerable metacestode damage, and further dose-response studies revealed that bortezomib (BTZ), a proteasome inhibitor developed for the chemotherapy of myeloma, displayed high anti-metacestodal activity with an EC_50_ of 0.6 µM. BTZ treatment of *E. multilocularis* metacestodes led to an accumulation of ubiquinated proteins and unequivocally parasite death. In-gel zymography assays using *E. multilocularis* extracts demonstrated BTZ-mediated inhibition of protease activity in a band of approximately 23 kDa, the same size at which the proteasome subunit beta 5 of *E. multilocularis* could be detected by Western blot. Balb/c mice experimentally infected with *E. multilocularis* metacestodes were used to assess BTZ treatment, starting at 6 weeks post-infection by intraperitoneal injection of BTZ. This treatment led to reduced parasite weight, but to a degree that was not statistically significant, and it induced adverse effects such as diarrhea and neurological symptoms. In conclusion, the proteasome was identified as a drug target in *E. multilocularis* metacestodes that can be efficiently inhibited by BTZ in vitro. However, translation of these findings into in vivo efficacy requires further adjustments of treatment regimens using BTZ, or possibly other proteasome inhibitors.

## Author Summary

Tapeworms (cestodes) are a class of important human pathogens, causing very severe diseases in man such as alveolar echinococcosis (*Echinococcus multilocularis*), cystic echinococcosis (*E. granulosus*) and neurocysticercosis (*Taenia solium*). Current treatments are mainly based on benzimidazoles that show some limited activity against cestode larvae, but often do not kill them. These compounds have to be taken for extended periods of time, and can cause adverse side-effects. Cestode infections cause neglected diseases and the pharmaceutical industry is generally not interested in investments for developing novel bioactive compounds. In this study we focus on a panel of FDA-approved drugs and assessed them in *E. multilocularis*, which causes the most deadly of all helminth infections. One compound, the anti-cancer drug bortezomib, exhibits considerable in vitro activity against *E. multilocularis* metacestodes, and we provide evidence that it acts on the proteasome. In experimentally infected mice bortezomib activity was lower than the currently used albendazole and induced adverse effects. Bortezomib is therefore not a useful drug for treatment of *Echinococcus* larvae, but our results demonstrate that in future studies the cestode proteasome should gain more attention as a drug target.

## Introduction

Parasitic tapeworms (*Cestoda*) such as *Taenia solium*, *Echinococcus granulosus* and *Echinococcus multilocularis* are important human pathogens causing neglected diseases. In total they are estimated to account for 22–55 million disability-adjusted life years (DALYs) and thereby are comparable to the leading parasitic disease malaria (39 million DALYs) [1]. All three species grow as adult tapeworms in the intestine of their final host, where eggs are produced upon fertilization. After fecal shedding, the eggs contaminate the environment, and oral uptake leads to infections of intermediate hosts such as accidentally humans. Whereas adult *Echinococcus* spp. do not affect their final host, *Taenia* spp. infection in humans can lead to mild gastrointestinal symptoms such as diarrhea and abdominal pain [2]. In their intermediate host, tapeworms form the larval metacestode stage, which causes severe diseases in humans such as neurocysticercosis (NC; caused by *T. solium*), cystic echinococcosis (CE; caused by *E. granulosus*) and alveolar echinococcosis (AE; caused by *E. multilocularis*). Besides surgical removal of the parasites, which is not always possible, other treatment options include drug-based therapy using praziquantel and albendazole (ABZ) against NC, and ABZ and mebendazole against AE and CE [2], [3]. These drugs, however, were not specifically developed for the treatment of cestode infections, but rather represent general anthelminthics that are currently also used against cestodes. While these compounds have clearly improved the life expectancy and living conditions of affected patients, novel therapeutic options are needed due to the fact that there are cases where the drugs in use have proven ineffective, toxicity has been reported, and/or resistance formation has occurred [2], [3].

For the identification of novel potential treatment options against cestode infections, *E. multilocularis* represents the prime model parasite. This is due to the fact that a metacestode culture system allowing the long-term maintenance, proliferation and differentiation of the larval stage has been developed, which permits detailed studies of drug-mediated effects [4]–[6]. In addition, comprehensive information on the *E. multilocularis* genome and transcriptome has been made publically available, and advanced molecular biological techniques such as stem cell cultivation and RNAi have been established [7], [8].

AE, caused by *E. multilocularis*, represents the most deadly of all helminth infections, with an estimated 18'235 new cases per year, and the vast majority (over 90%) occurring in China [9]. European countries such as Switzerland, France and Germany are regarded as hot-spots of endemicity, and within Europe an average number of 168 new cases per year have been reported [9]. Although AE is rare, the severity of the disease results in an estimated 600'000 DALYs, which renders the impact of AE comparable to tropical diseases such as leprosy, dengue and schistosomiasis [1]. Upon infection, metacestodes grow and proliferate similar to a malignant tumor, mainly in the liver. Metastases formation often occurs close by or at more distant parts of the body. The continuous and infiltrative growth of metacestodes causes loss of function of affected organs. AE is fatal if not treated appropriately. Currently applied chemotherapeutical treatment options are not parasitocidal, but parasitostatic [3]. Thus, patients have to undergo chemotherapy for extended periods of time, often lifelong, in order to avoid further growth and spread of metacestode tissue.

A limited number of compounds were found to be active against *E. multilocularis* metacestodes in vitro, including nitazoxanide and derivatives [10], [11], 2-methoxyestradiol [12], artesunate and semi-synthetic derivatives [13], mefloquine and enantiomers [14], [15], dicationic compounds [15], [16], imatinib [17], ruthenium(II) phosphite complexes [18], and synthetic amino-ozonides [19]. For two compounds, the di-cationic diguanidino compound DB1127 and mefloquine, profound anti-parasitic activities were also detected in experimentally infected mice [14], [16]. Unfortunately, the lack of financial incentives and the technical difficulties associated with working with *Echinococcus* parasites have hampered efforts to develop more effective treatment against AE. We therefore changed our focus towards drugs that are already approved and marketed and that will easier find a way to application. We have earlier developed a screening assay for the identification of novel compounds that act against *E. multilocularis* metacestodes, which is based on the detection of phosphoglucose isomerase (PGI) activity in medium supernatants of in vitro-cultured metacestodes [10]. PGI is a prominent component of metacestode vesicle fluid, which is released upon damage by physical or chemical means. In this study we employed the PGI assay to screen a library of 426 FDA-approved drugs for in vitro effects on metacestodes. Our initial screen at 20 µM yielded 7 compounds with negative impact on *E. multilocularis* metacestode viability, many of which had well-defined mechanisms of action, thus making them useful tools to study basic biology in addition to being potential therapeutics. Of these compounds, bortezomib (BTZ), a proteasome-inhibitor in use for treatment of multiple myeloma [20] and mantle cell lymphoma [21], was further characterized. We here demonstrate that the proteasome represents a valid cestode drug target. Finally we also assessed the applicability of BTZ in vivo by employing the mouse model for secondary AE.

## Materials and Methods

All chemicals were purchased from Sigma (St. Louis, MO), if not stated otherwise. Cell culture reagents were from Gibco-BRL (Zürich, Switzerland), with the exception of Dulbecco's modified Eagle medium (DMEM) and fetal bovine serum (FBS) that were ordered from Biochrom (Berlin, Germany). A commercially available FDA-approved drug library containing 426 compounds was purchased from Selleck Chemicals (Houston, TX), and Bortezomib was obtained from Mobitec (Göttingen, Germany).

### In vitro culture and drug screening of *E. multilocularis* metacestodes

*E. multilocularis* metacestode tissue material was retrieved from euthanized BALB/c mice that had been intraperitoneally infected with *E. multilocularis* metacestodes (isolate H95). Dissected parasite material was prepared as described earlier [4]. In vitro-cultured metacestode vesicles were used for experimental studies after more than 6 weeks of in vitro cultivation when they were 2 to 4 mm in diameter [10].

FDA-library drugs were supplied as 10 mM stock solutions in DMSO and were prediluted in medium 1∶10 before use. Initial screening on metacestodes was performed at 20 µM in singlets and activity was defined as 50% or more of the internal control DB1127 at the same concentration. Morphological effects on vesicles were also checked in parallel. Further assessments of compounds that were active in the initial screen were done in triplicates at 20, 10, 5, 1 and 0.1 µM (and in addition 0.05 and 0.01 µM for EC_50_ calculation of BTZ) and averages as well as standard deviations calculated in Microsoft Excel 2010. The protocol described by Stadelmann et al was applied [10], which is based on the release of PGI from metacestodes that are damaged during treatment. Negative controls contained the corresponding amounts of DMSO. As a positive control DB1127 (20 µM) [15], [16] was applied. PGI measurements were performed as described earlier [10], with the difference that a 2300 EnSpire multilabel reader (Perkin-Elmer, Turku, Finland) was used for measurement of increase in absorbance at 340 nm. PGI activity was calculated in Microsoft Office Excel 2010 from the linear regression of the enzyme reaction over time and set into relation to the positive control DB1127. The activity of the DMSO control was subtracted.

### Scanning (SEM) and transmission electron microscopy (TEM)

In vitro-cultured *E. multilocularis* metacestodes were treated with BTZ at concentrations of 0.01, 0.05, 0.1 and 0.5 µM for a period of 5 days at 37°C, 5% CO_2_. They were washed once in PBS and were fixed in 2.5% glutaraldehyde in 100 mM sodium cacodylate buffer (pH 7.2) for 2 h, followed by post-fixation in 2% OsO_4_ in 100 mM sodium cacodylate buffer for 2 h. Subsequently, specimens were washed in distilled water, treated with 1% uranyl acetate for 30 min, washed again in water, and dehydrated by sequential incubations in ethanol (30–50–70–90–100%). For SEM analysis, dehydrated specimens were finally immersed in hexamethyl-disilazane and air dried under a fume hood. They were then sputter-coated with gold and inspected on a JEOL 840 scanning electron microscope operating at 25 kV. For TEM analysis, fixed and dehydrated specimens were embedded in Epon 812 resin (Fluka) and polymerisation was carried out at 65°C overnight. Sections were cut on a Reichert–Jung ultramicrotome (Reichert–Jung, Vienna, Austria), loaded onto 300-mesh copper grids (Plano GmbH, Marburg, Germany), stained with uranyl acetate and lead citrate, and were viewed on a Philips 400 transmission electron microscope (Philips Electron Optics, Eindhoven, Holland) operating at 80 kV.

### Sequence analyses

The *E. multilocularis* proteasome subunit beta 5 gene (*psmb5*) and the proteasome subunit beta 1 gene (*psmb1*) were identified by BLAST on the website www.genedb.org/Homepage/Emultilocularis. Amino acid sequence analyses of PSMB5 of *E. multilocularis* (GeneDB EmuJ_000590200_1, referred to as EmPSMB5), *Homo sapiens* (UniProtKB/Swiss-Prot P28074) and *Saccharomyces cerevisiae* (UniProtKB/Swiss-Prot P30656) were performed by alignment in ClustalW 2.1 and they were further analyzed based on the identified binding sites and active residues of PSMB5 as described by Gille et al and Groll et al [22], [23].

### Relative quantification of ubiquitinated proteins by western blot

Ubiquitinated proteins in metacestodes were detected and quantified by Western blot. In short, metacestode vesicles were incubated in DMEM in the presence or absence of 10 µM BTZ for 24 h at 37°C. Assays were carried out in triplicates. Vesicles were washed in PBS, physically broken by pipetting, and vesicle fluid was removed after centrifugation. After another wash in PBS, metacestode tissue was taken up in RIPA (50 mM Tris-HCl (pH 7.4), 1 mM EDTA, 150 mM NaCl, 0.1% SDS, 0.5% doc, 1% Tx-100) and incubated on ice for 40 min. Following centrifugation for 30 min, at 4°C and 16'100× g, the protein concentration in the supernatant was determined by BCA protein assay kit. 15 µg of each sample were separated by 12% SDS-PAGE, blotted onto nitrocellulose, and Western blots were incubated in Odyssey blocking solution (Licor Biosciences GmbH, Bad Homburg, Germany) +0.1% Tween for 2 h. The primary antibodies were applied overnight at 4°C in Odyssey blocking solution (mouse anti-ubiquitin, 1∶500, sc-8017 (Santa Cruz Biotechnology, Heidelberg, Germany); rabbit anti-GAPDH, 1∶1'000, ab36840 (Abcam, Cambridge, UK)). Secondary antibodies were applied for 1 h at room temperature in Odyssey blocking solution (anti-rabbit IR680RD 680, 1∶10'000, Licor 926-68071; anti-mouse IR800CW, 1∶10'000, Licor 926-32210, Licor Biosciences, Bad Homburg, Germany). After washing in PBS, proteins were visualized in an Odyssey Scanner (Licor). Semiquantitative analysis of signals was conducted in ImageJ Software 1.47 and Microsoft Office Excel 2010.

### Proteasome activity assay

Inhibitory effects of BTZ on the proteasome and chymotrypsin-like proteases of *E. multilocularis* metacestode extracts were assessed as described in Qiu et al [24]. In vitro-cultured *E. multilocularis* metacestodes were washed in PBS and disrupted with a 1 mL pipette. After further washing in PBS, vesicle tissue was resuspended in proteasome buffer (1 M Tris-HCl pH 7.4, 1 mM EDTA, 2 mM ATP, 20% glycerol, 4 mM DTT) on ice and sonicated at 4°C for 1 min in a Q700 Sonicator (Q Sonica, Newtown, CT, USA) (Amplitude 20, alternating on/off cycles of 5 seconds). The cell extract was centrifuged at 16'100× g for 10 min at 4°C and the protein content of the supernatant was measured by Pierce BCA protein assay kit (Thermo Scientific, Waltham, MA, USA). The proteasome activity assay was performed in 96-well plates in triplicates, containing 150 µg of cell extract each, and BTZ (solubilized in DMSO) was added (final BTZ concentrations 100, 10, 1, 0.1, 0.01, 0.001 µM). DMSO was added as a negative control, and incubations lasted for 1 h at 37°C. In some experiments, Halt protease inhibitor cocktail (Thermo Scientific) was added prior to addition of the fluorogenic substrate N-Succinyl-Leu-Leu-Val-Tyr-7-amino-4-methylcoumarin (SLLVT-AMC, in 0.05 M Tris-HCl pH 8.0, 0.5 mM EDTA) to a final concentration of 40 µM. Fluorescence (excitation at 380 nm and emission at 440 nm) was measured over 120 min in an EnSpire multilabel reader. Relative chymotrypsin-like activity was calculated via the regression of product formation over time and expressed as percentage of the DMSO control using Microsoft Excel 2010.

### Fluorescent zymography

Fluorescent zymography was performed basically as described earlier [25]. Metacestode tissue extracts were suspended in non-reducing sample buffer. Metacestode extracts (25 µg per lane) and a positive control (bovine chymotrypsin, 5 ng per lane) were separated by 12% SDS-PAGE (with only 0.075% SDS in the stacking gel), after which the gel was washed two times for 30 min in SDS-removal solution (2.5% Tx-100), followed by several wash steps in water. Subsequently the gel was immersed in 0.05 M Tris-HCl (pH 7.4), 10 mM CaCl_2_, 0.005% Tx-100, 200 µM SLLVT-AMC, either in the presence of 20 µM BTZ or the corresponding DMSO control. After incubation for 45 min at 37°C, the fluorescent signal (365 nm) representing chymotrypsin activity was detected on a transilluminator (Syngene, Cambridge, UK). For the visualization of the marker, the gel was subsequently silver stained and the markers transferred to the gel image in Adobe Illustrator CS 11.0.

For detection of EmPSMB5 by Western blot, metacestode extracts were prepared as described above and loaded on a 12% SDS-PAGE at 100 µg per lane in non-reducing sample buffer. Western blotting was basically performed as described above by use of infrared-dyed conjugate antibodies and detection employing the Odyssey scanner (1^st^ antibody: anti-PSMB5, 1∶1'000, SAB2101895; 2^nd^ antibody: goat anti-rabbit IR680RD 680, 1∶10'000).

### In vivo study on the efficacy of BTZ treatment in mice experimentally infected with *E. multilocularis* metacestodes

Experiments were performed according to Swiss Animal protection law. 40 female BALB/c mice (source: Charles River, Sulzfeld, Germany), 8 weeks of age with an average body weight of 20–25 g, were kept in a temperature-controlled 12/12 hours light cycle room with food and water *ad libitum*. For infection, in vitro-cultured metacestodes were broken with a 1 mL pipette tip, centrifuged at 500×g for 5 min at room temperature, and the resulting pellet was resuspended in an equal volume of PBS. All mice were infected intraperitoneally (i.p.) with 200 uL of the metacestode suspension. During the following 6 weeks, the mice were adapted to honey feeding (Blütenhonig M-Budget, Migros, Switzerland diluted 1∶1 in 1% carboxymethyl cellulose ( = honey/CMC)) twice a week as described [26]. At 6 weeks post-infection, mice were randomly distributed into 8 cages with 5 animals each. Each treatment group consisted of 2 cages. Group 1: negative control, received 100 µL honey/CMC suspension orally each day and 100 µL of PBS/CMC with 2.8 µL DMSO injected i.p. once per week. Group 2: albendazole control, received 200 mg/kg albendazole (ABZ) in honey/CMC orally on a daily basis and 100 µL of PBS/CMC with 2.8 µL DMSO injected i.p. once per week. Group 3: BTZ treatment, received 100 µL honey/CMC suspension by daily oral application and 0.7 mg/kg BTZ in 100 µL PBS/CMC once per week. Group 4: combined ABZ and BTZ treatment, received 200 mg/kg ABZ in honey/CMC orally each day and 0.7 mg/kg BTZ in 100 µL PBS/CMC once per week. The described treatment was pursued for the first 3 weeks. After that, the treatment regime was adapted to a BTZ dosage of 0.5 mg/kg, but administered twice per week. This treatment was performed for another 3 weeks. All other treatments remained the same. After a total treatment duration of 6 weeks, mice were euthanized by CO_2_. All parasite tissue was collected after necropsy and the total parasite weight per mouse measured. To evaluate data distribution, Shapiro-Wilk test was applied in the software R version 3.0.1 (R core team, A Language and Environment for Statistical Computing, R Foundation for Statistical Computing, Vienna, 2013). Further, the data was analyzed by one-way ANOVA and Bonferroni-adjusted P values calculated by Pairwise T-Test in R. Data was visualized by boxplot in Microsoft Office Excel 2010.

### Ethics statement

The in vivo study was approved by the Animal Welfare Committee of the Canton of Berne under the licence number BE 103/11. The study was performed in compliance with the Swiss animal protection law (TschV, SR 455).

## Results

### In vitro screening of a FDA-approved drug library identifies high activity of BTZ against *E. multilocularis* metacestodes

**Figure 1:**
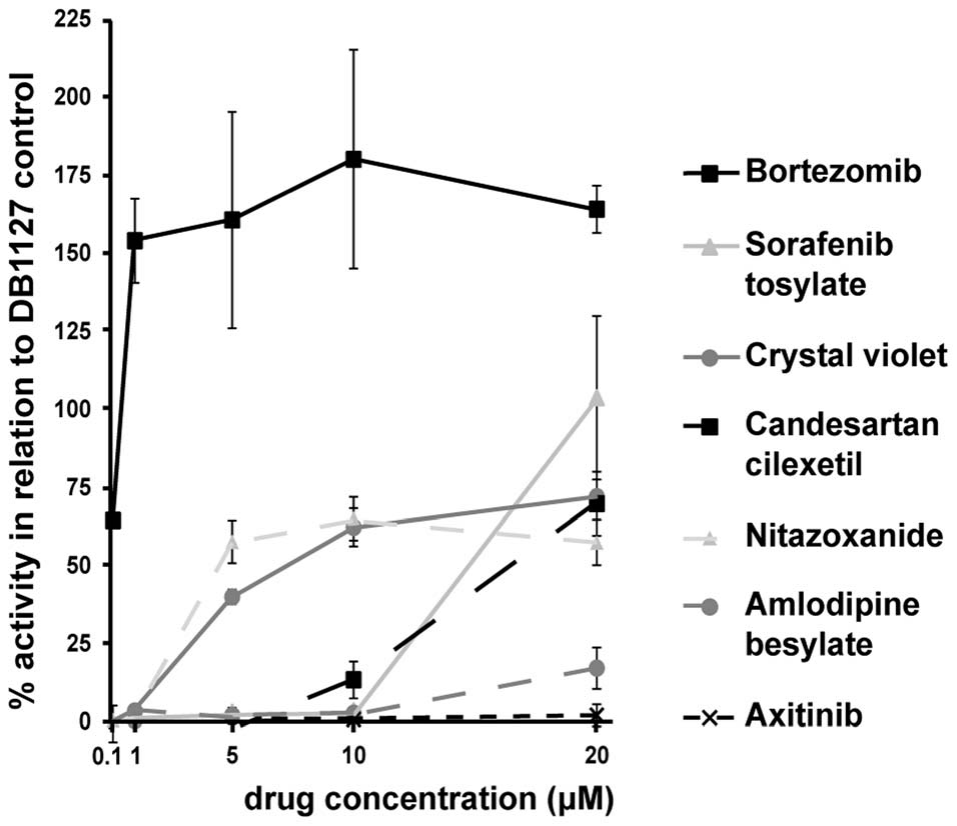
Activity of the most active drugs of the FDA library against *E. multilocularis* metacestodes in vitro. The seven most active drugs (more than 50% damage of the control DB1127 at 20 µM after 5 days; BTZ, sorafenib tosylate, crystal violet, candesartan cilexetil, nitazoxanide, amlodipine besylate and axitinib) of the FDA drug library screen were further tested in triplicates by PGI assay at different concentrations (20, 10, 5, 1, 0.1 µM) and for their anti-metacestode activity in vitro. As a positive control, DB1127 was applied at 20 µM and the different drug activities are expressed as percentage of the positive control. DMSO served as a negative control at same dilutions as the drugs and was subtracted from all values. Note the high activity of BTZ down to the concentration of 0.1 µM.

A library of 426 FDA-approved drugs was screened employing the metacestode based PGI assay, using an initial concentration of 20 µM and a treatment duration of 5 days. The complete results of this screening are shown in Table S1. Seven of these drugs exhibited more than 50% of the activity of DB1127, one of the current lead compounds [15], [16] and this was also confirmed by macroscopical observations of the vesicles. The 7 active compounds were further evaluated in a concentration series (20, 10, 5, 1, 0.1 µM) (Figure 1). Axitinib and amlodipine besylate turned out to be wrong-positive hits in the initial screen and no longer showed activity of more than 50% when tested at 20 µM in triplicates. Five compounds (BTZ, sorafenib tosylate, crystal violet, candesartan cilexetil and nitazoxanide) were shown to be still active at 20 µM, but for sorafenib tosylate and candesartan cilexetil the activity was lost already at 10 µM. The other three compounds, nitazoxanide, crystal violet and BTZ, retained their anti-parasitic activity at lower concentrations (Figure 1). Crystal violet was not further pursued, since it is a topical disinfectant that cannot be used for internal drug application [27]. Nitazoxanide was also not followed-up, as this compound had been already extensively studied before and did not show beneficial effects in human patients [11], [28]. BTZ emerged as the most promising compound, showing high anti-metacestode activity at concentrations below 1 µM, with a calculated EC_50_ of 0.6 µM.

### Bortezomib treatment of *E. multilocularis* metacestodes induces severe morphological and ultrastructural changes

**Figure 2:**
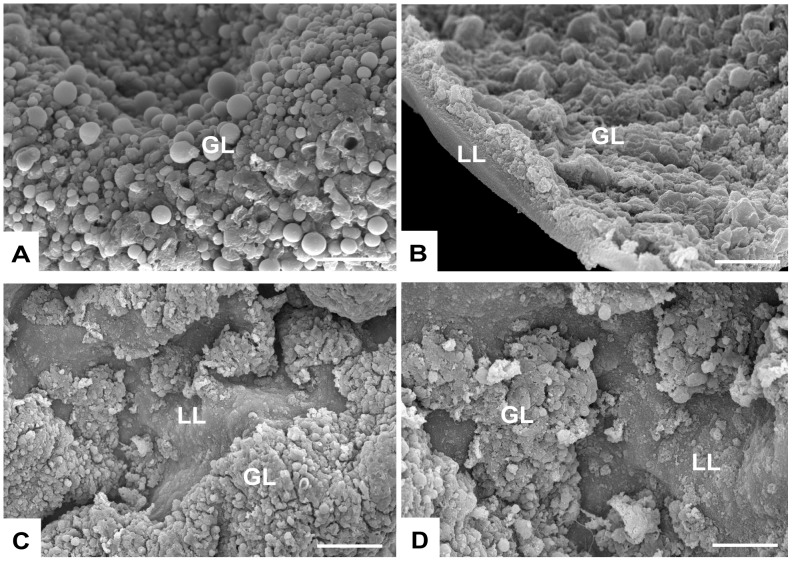
Scanning electron microscopical (SEM) assessment of BTZ-treated metacestodes. *E. multilocularis* metacestodes were treated with the solvent DMSO (A), or with 0.01 µM (B), 0.05 µM (C) or 0.5 µM (D) BTZ for 5 days, and were processed for SEM. Note the distorted morphology of the germinal layer (GL) in those specimens treated with 0.05 and 0.1 µM BTZ. Bars = 250 µM.

*E. multilocularis* metacestodes were subjected to 5 days BTZ treatment at concentrations ranging between 0.01 and 0.5 µM, and the effects were visualized by SEM (Figure 2). Metacestodes are basically fluid-filled vesicles that are composed of an outer acellular laminated layer that covers the entire surface of the parasite, and an inner germinal layer that represents the living parasite tissue. Treatment with 0.01 µM BTZ did not have a dramatic effect on the morphology of the germinal layer (Figure 2B). However, more pronounced morphological alterations were observed after treatment with 0.05 µM BTZ and higher (Figure 2C, D), which lead to partial separation of the parasite tissue from the inner surface of the laminated layer. In contrast, control incubations in the presence of the corresponding amount of DMSO did not lead to any alterations and metacestodes exhibited an intact germinal layer with normal morphological features (Figure 2A).

**Figure 3:**
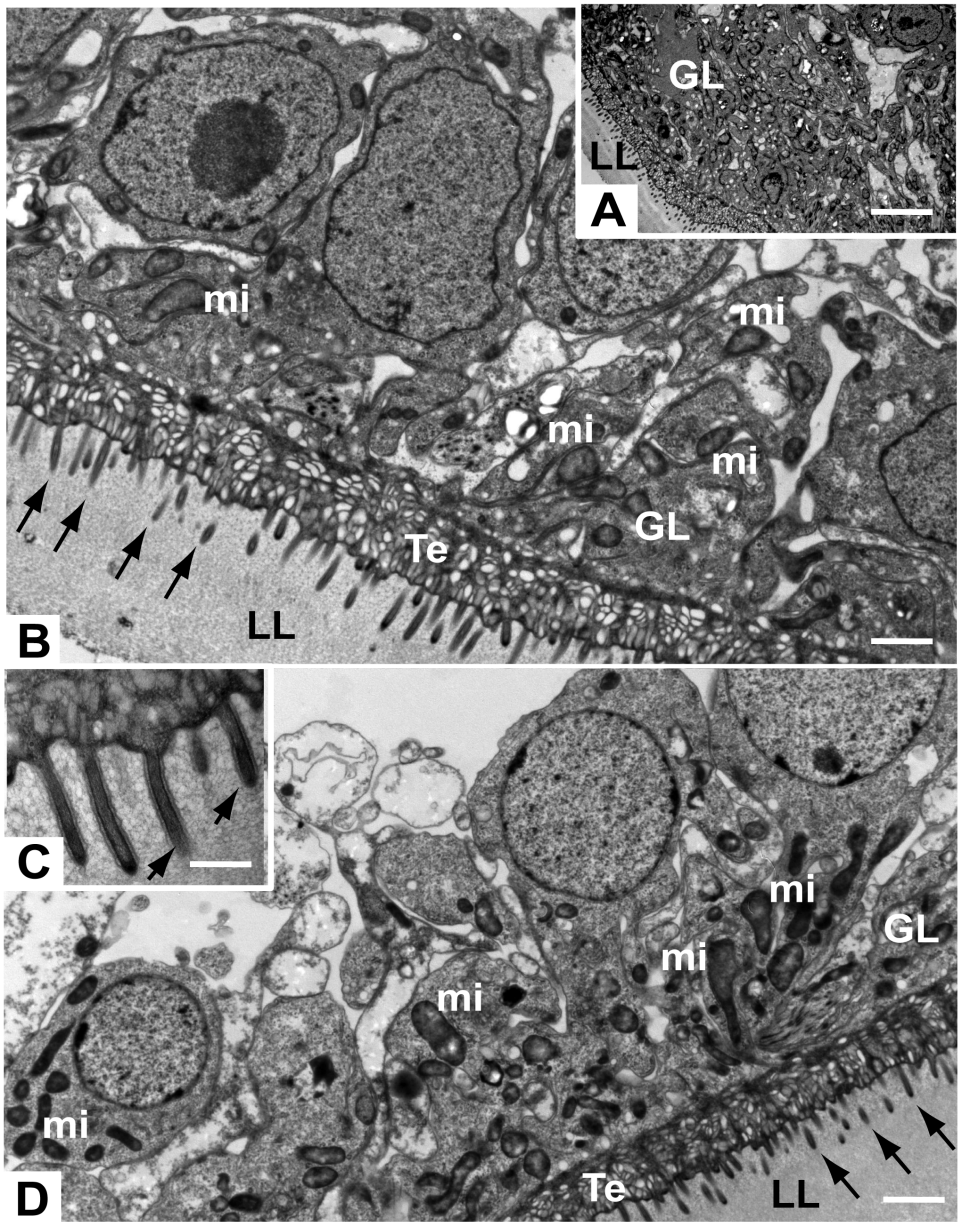
Transmission electron microscopy (TEM) of untreated and BTZ-treated *E. multilocularis* metacestodes. Parasites were treated with the solvent DMSO as a control (A, B) or with 0.01 µM (C, D) BTZ for 5 days, and were processed for TEM. (A, B) show metacestodes maintained in the absence of BTZ at low (A) and higher (B) magnification. The most outer laminated layer (LL), the tegument (Te) and the inner germinal layer (GL) are clearly discernible; mi = mitochondria. Black arrows point towards microtriches that originate at the tegument and protrude into the LL. In parasites treated with 0.01 µM BTZ no alterations are evident (C, D). Undifferentiated cells and mitochondria exhibit a similar morphology as the controls, microtriches (black arrows) are still intact. (C) shows a higher magnification view of microtriches. Bars in A = 4 µm; B = 1.6 µm; C = 0.5 µm; D = 1.6 µm.

**Figure 4:**
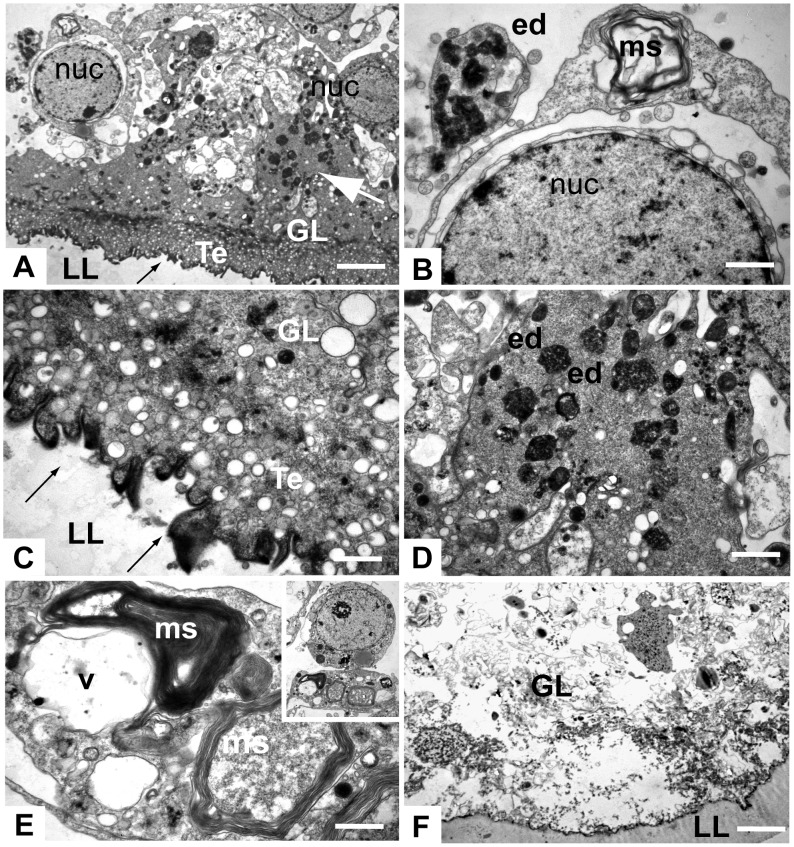
Transmission electron microscopical (TEM) assessment of BTZ-treated metacestodes. *E. multilocularis* metacestodes were treated with 0.05 (A–E) or 0.5 µM (F) BTZ for 5 days, and were processed for TEM. (A) shows an overview of a section through the metacestode wall after treatment with BTZ at 0.05 µM, (B–F) are higher magnification views of selected areas in (A). Profound changes occurred at the interface between tegument (Te) and laminated layer (LL), with evident microtriches distortion (black arrows, A, C). The cytoplasm of germinal layer cells is filled with electron dense deposits (marked by white arrow in (A) and designated as ed in (B) and (D)), and often vacuoles containing membrane stacks (ms) resembling authophagosomes are visible (B, E and insert in E). Nuclei (nuc) appear still largely intact (A, B). Treatment with 0.5 µM BTZ results in completely distorted GL and only residual LL (F). Bars in A = 2.1 µm; B = 0.5 µm; C = 0.7 µm; D = 0.7 µm; E = 0.35 µm; F = 3 µm.

Investigations using TEM confirmed these findings. Sections through control metacestodes showed an intact germinal layer, composed of numerous cell types that form the parasite tissue (Figure 3A, B). The most distal part of the parasite tissue is composed of the tegument, a syncytial layer that surrounds the entire metacestode, with numerous microtriches protruding into the laminated layer. The adjacent metacestode tissue is composed of undifferentiated cells with a large nucleus and nucleolus (also described as germinative or stem cells), and differentiated cell types such as glycogen storage cells, muscle cells, nerve cells and connective tissue (Figure 3A, B). Treatment with 0.01 µM BTZ did not notably affect the ultrastructure of the germinal layer (Figure 3C, D). However, treatment with 0.05 µM had a major impact, with parasites exhibiting clear signs of apoptosis (Figure 4). Overall, BTZ treatment was followed by a distinct degeneration of the tegumental structure and in particular of the microtriches (Figure 4A, C). In many parts, however, the cellular membranes were still intact, and also in undifferentiated cells the nucleus appeared to be seemingly unaffected, while the cytoplasm was filled with distinct vacuoles containing membrane stacks and/or electron-dense cytoplasmic inclusions, which were situated most likely within the former mitochondria (Figure 4A, B and D). In many instances, cells were harboring large cytoplasmic vacuoles filled with membranous material that structurally resembled autophagosomes (Figure 4B, E). Treatment with 0.5 µM BTZ had an even more dramatic effect, and resulted in complete destruction of the germinal layer within the 5 days of incubation (Figure 4F).

### Sequence analysis of EmPSMB5

**Figure 5:**
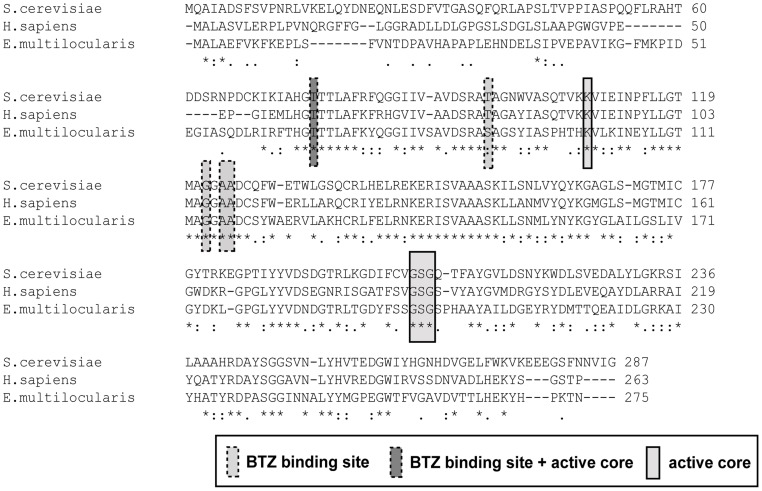
Amino acid sequence alignment of the proteasome subunit 5 beta of *Saccharomyces cerevisiae, Homo sapiens* and *E. multilocularis*. The amino acids of the active core of PSMB5 are highlighted in light grey, amino acids involved in BTZ binding are highlighted in dark grey (based on [22], [23]). One amino acid is both part of the active core and involved in BTZ binding (T67 of *E. multilocularis* sequence). One amino acid involved in BTZ binding shows a conservative replacement (T→S88). The *E. multilocularis* PSMB5 was 52% identical to *S. cerevisiae* PSMB5 and 61% identical to the human ortholog. The alignment was performed in ClustalW2.1.

In human patients, the anti-cancer drug BTZ inhibits the proteolytic activity of the proteasome subunit beta 5 (PSMB5), and, to a lesser extent, also the beta 1 subunit (PSMB1) [29]. In *E. multilocularis* we identified proteins ortholog to PSMB5 and PSMB1: EmuJ_000590200_1 (referred to as EmPSMB5) and EmuJ_000252500_1 (referred to as EmPSMB1). For further analysis we focused on the EmPSMB5 subunit since it is predominantly inhibited by BTZ [29]. PSMB5 sequence alignments from *E. multilocularis*, *H. sapiens* and *S. cerevisiae* showed that the *E. multilocularis* ortholog shares 61% identity with *H. sapiens* and 52% identity with *S. cerevisiae* orthologs on the amino acid level (Figure 5). The amino acids needed for proteolytic function of this proteasome subunit are present in EmPSMB5 (Figure 5). In addition, the amino acids involved in binding of BTZ in human PSMB5 are also present in EmPSMB5 with one residue being changed (Serine 88 instead of Threonine, see Figure 5). Thus the described molecular target of BTZ in humans is also present in *E. multilocularis*.

### BTZ is targeting the *E. multilocularis* proteasome

**Figure 6:**
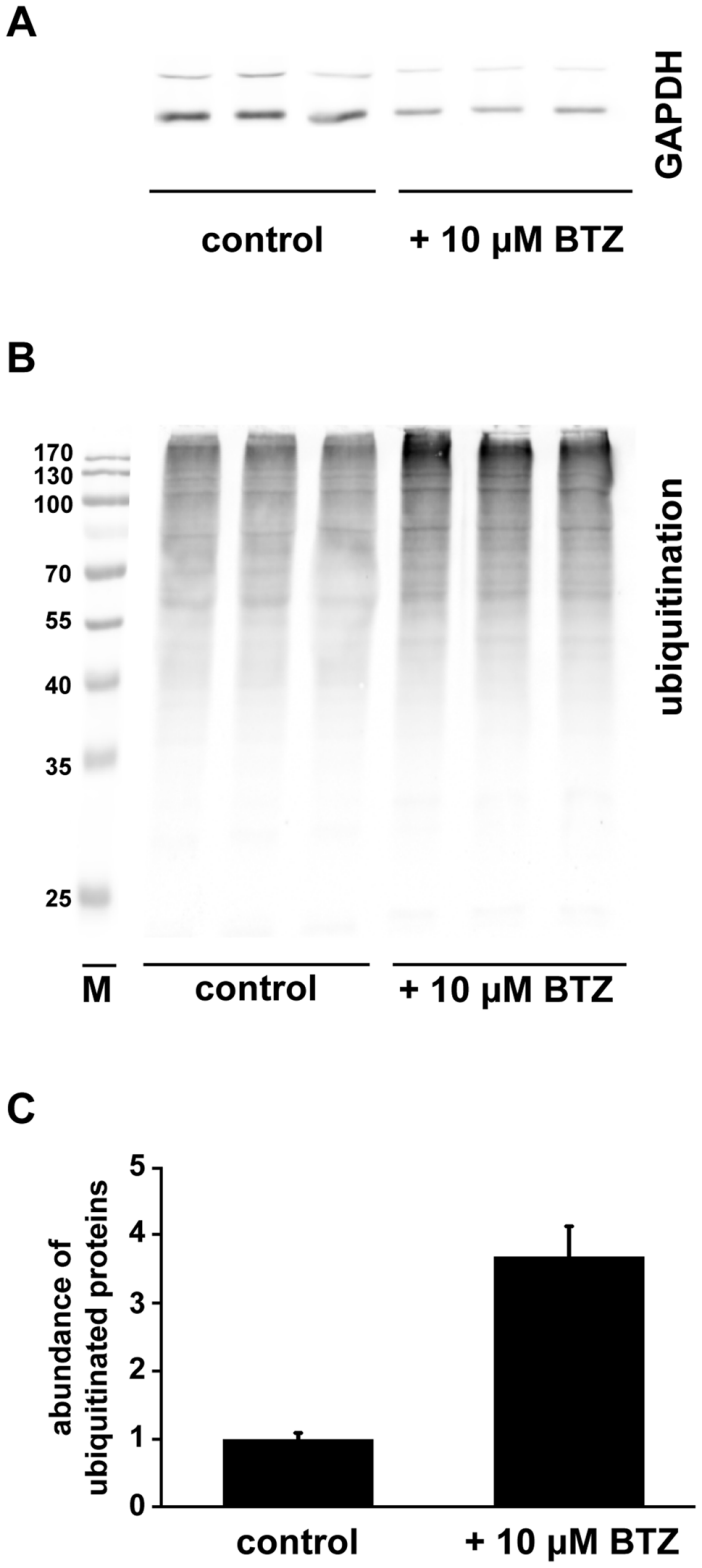
Accumulation of ubiquitinated proteins upon *E. multilocularis* metacestode treatment with BTZ. *E. multilocularis* metacestodes were treated in vitro with BTZ (10 µM) for 24 h in triplicates and proteins extracted and visualized by Western blot (A, B). Treatment with equal amounts of DMSO served as a negative control. GAPDH was used as sample loading control (A), the anti-ubiquitin antibody detected ubiquitinated proteins in each sample; M = Marker (B). Semiquantitative analysis of ubiquitinated proteins was performed in Image J and relative protein abundance shown in relation to the untreated controls (C). This experiment was performed twice and lead to similar results of 3.5 times accumulation of ubiquitinated proteins upon BTZ treatment.

**Figure 7:**
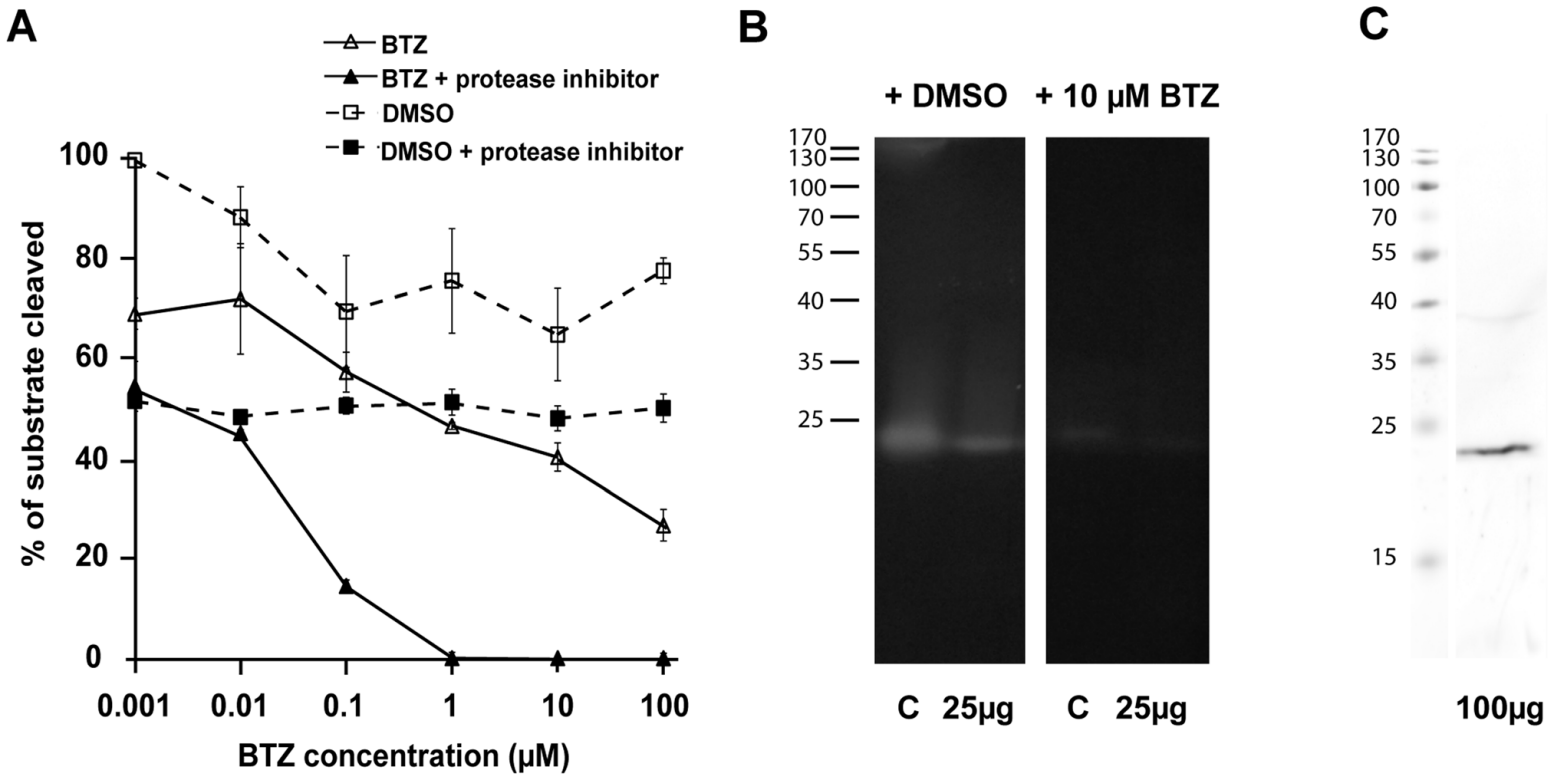
Proteasome activity in *E. multilocularis* cell extracts can be inhibited by BTZ. Chymotrypsin-like activity of the *E. multilocularis* proteasome was shown in cell extracts from in vitro-cultured metacestodes by applying the fluorogenic substrate SLLVT-AMC in solution (A) and in gel (B). Addition of BTZ led to dose-dependent inhibition of this activity. A, in solution proteasome assay with and without Halt protease inhibitor (to inhibit other proteases). As a control DMSO was added to same amounts as with the drug and the activity calculated as percentage from the DMSO control. This experiment was performed in technical quadruplicates and was repeated three times, which led to similar results each time. B, in gel assay to detect chymotrypsin-like activity in *E. multilocularis* cell extracts. Bovine chymotrypsin was used as positive control (C, 5 ng per lane), *E. multilocularis* extract was loaded at 25 µg. On the left the gel was incubated with DMSO, on the right with BTZ at 10 µM. C, Western blot was performed to detect the *E. multilocularis* proteasome subunit beta 5 (EmPSMB5). Note that this protein migrates at the same apparent molecular mass as the active band in B that could be inhibited by BTZ. Experiments B and C were repeated both twice leading to the same results.

BTZ was developed as an anti-cancer drug that would target the proteasome, and thus induces apoptosis of tumor cells [20]. While TEM suggested that BTZ treatment of metacestodes resulted in features resembling apoptotic cells, we investigated whether BTZ also induced accumulation of ubiquitinated proteins, which represents another hallmark of proteasome inhibition. Ubiquitination levels in BTZ-treated and control metacestodes were followed in relation to GAPDH as a reference protein by Western blot, and treatment with 10 µM BTZ for 24 h led to a significant accumulation of ubiquitinated proteins (Figure 6). To rule out that this was due to an increased activity of ubiquitinating enzymes, the proteolytic activity of the proteasome was assessed. The fluorogenic molecule SLLVT-AMC was used as a substrate for chymotrypsin-like proteases (such as PSMB5). Upon addition of BTZ to *E. multilocularis* vesicle extract, a concentration-dependent reduction in substrate cleavage was observed (Figure 7A). In order to block other proteases that show chymotrypsin-like activity, Halt protease-inhibitor cocktail, that could also inhibit parts of the proteasome [30], was added. This led to a more stable DMSO control and to complete absence of substrate cleavage at BTZ concentrations of 1 µM and higher. The IC_50_ of BTZ in *E. multilocularis* metacestode extracts without Halt protease inhibitor was calculated to be 0.76 µM, and at 1 µM BTZ reduced substrate cleavage by 38.4±0.9% as compared to the DMSO control (Figure 7A). Inhibition of proteases by Halt protease inhibitor led to a reduction of substrate cleavage by 32±3.4%, and the combined BTZ and protease inhibitor treatment completely abolished chymotrypsin-activity.

Inhibition of chymotryptic protease activity in vesicle extracts by BTZ was further visualized after separation of the vesicle extract by non-reducing SDS-PAGE and exposure of the gel to the same substrate SLLVT-AMC. One clear chymotryptic active band was visible at around 23 kDa (Figure 7B). Upon addition of 10 µM BTZ this signal was strongly inhibited. Western blot analysis revealed that EmPSMB5 had the same apparent molecular mass as the chymotryptic active band from the parasite extract (Figure 7C). In conclusion, BTZ has a strong impact on the functionality of the proteasome of *E. multilocularis*.

### Preliminary assessment of BTZ treatment in mice secondarily infected with *E. multilocularis* metacestodes reveals limited in vivo activity

**Figure 8:**
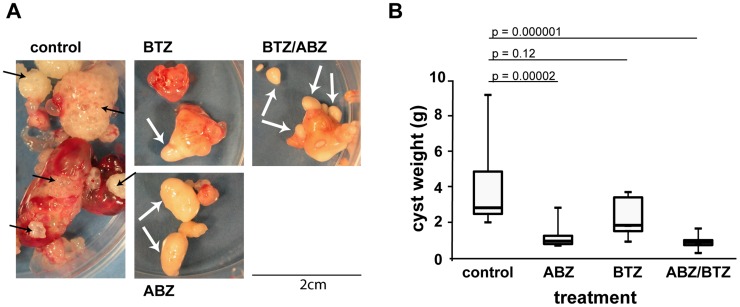
In vivo treatment of secondary *E. multilocularis* infected Balb/c mice with BTZ reduces parasite weight. Balb/c mice were i.p. infected with in vitro-cultured *E. multilocularis* parasite material. After 6 weeks, treatment was performed for 6 weeks (5 days a week) with 2×5 mice in each treatment group. Controls received honey/CMC p.o. and PBS i.p. ABZ received 200 mg/kg ABZ in honey/CMC and PBS i.p. BTZ received honey/CMC p.o. and 0.7 mg/kg BTZ i.p. once a week for three weeks, then 0.5 mg/kg twice a week for another three weeks. ABZ/BTZ received ABZ p.o. and BTZ i.p. as stated above. After euthanization parasite material was resected (A) and weighed (B). A, macroscopical assessment showed proliferating metacestode masses with many big and small vesicles (see black arrows) in the control group. All treated groups showed less metacestodes, especially less proliferating ones and more white and encapsulated, small cysts (see white arrows). B, parasite weight visualized by boxplot. Statistical analysis of log-transformed data confirmed a highly significant parasite mass reduction with ABZ and ABZ/BTZ treatment.

The effects of BTZ treatment by i.p. injection were investigated in secondarily infected Balb/c mice. Albendazole (in honey/CMC) was applied p.o. as a positive control, and as a negative control mice were treated with honey/CMC p.o. and PBS i.p. In general, mild to heavy diarrhea was observed for all mice treated i.p. with BTZ on the first and second day after injection. In addition, BTZ-treated mice showed neurological symptoms such as slight ataxia and reduced fur care eventually. One mouse of the BTZ-treated group died during the course of the experiment. Due to these adverse effects of BTZ, treatment was adjusted to a dosage of 0.5 mg/kg instead of 0.7 mg/kg, which led to better health status in long term, though diarrhea was still observed. After another 3 weeks of treatment at lower BTZ concentrations, all mice were euthanized, carefully examined and total parasite weight determined. In the control group, proliferating metacestodes with many small vesicles and surrounding blood vessels were found. In contrast, BTZ treatment led to whitish and unilocular cysts, some of them being calcified (Figure 8A). ABZ and ABZ/BTZ-treated mice also harbored similar whitish, unilocular cysts, some of them being as small as a 1–3 mm in diameter only. Shapiro-Wilk test suggested that the measured cyst weights did not follow a normal distribution (W = 0.7428, P = 6.76E-07), but rather a log-normal distribution (W = 0.9685, P = 0.3366). One-way ANOVA analysis of log-transformed values proved a significant difference between the groups (p = 5.36E-07). Post-Hoc analysis by Pairwise T-Test with Bonferroni-adjustment showed that there was a highly significant reduction in the ABZ and ABZ/BTZ-treated group as compared to the control (p = 2.2e-05 and p = 1.3e-06, Figure 8B). BTZ treatment also led to a reduction in parasite weight, albeit results were not significant (p = 0.1224, Figure 8B). Thus, in vivo treatment with BTZ has a clear effect on the morphological appearance of recovered *E. multilocularis* tissue, but in terms of parasite weight no significant reduction compared to untreated controls was observed.

## Discussion

Chemotherapeutical treatment of AE relies on the application of the two benzimidazoles ABZ and MBZ, which have been shown to interact with beta-tubulin, and thus lead to distortion of the cytoskeletal organization and associated functions within *E. multilocularis* metacestodes. In this study, we characterized the in vitro and in vivo activity of the proteasome inhibitor BTZ and defined the proteasome as a drug target in this parasite. This could have important implications also with regard to other clinically relevant cestodes.

BTZ was identified through a metacestode in vitro screen of 426 FDA-approved drugs at an initial concentration of 20 µM. This is the first time that such an extended screening with hundreds of compounds against *E. multilocularis* has been performed. We hereby employed an established assay, which demonstrates the damaging impact of compounds by detection of PGI activity, an enzyme that is abundant in the vesicle fluid and is released upon distortion of the physical integrity of the parasite cyst. Further concentration series of the most active drugs were performed and showed that in particular 3 drugs were also very highly active at lower concentrations: BTZ, nitazoxanide and crystal violet. The current drug for treatment of AE, ABZ, was not identified as an active compound within our screen of the FDA library. The issue of false negative compounds staying undetected by employing the PGI assay has arisen before [31]. A likely explanation for the missing activity in vitro is that in vivo ABZ gets further metabolized to its active derivatives albendazolsulfoxide and albendazolsulfone. In addition, the impact of host immunity cannot be simulated through in vitro testing.

The activity of BTZ (EC_50_ of 0.6 µM in the PGI assay) is much higher compared to other drugs tested so far by the PGI assay (e.g. mefloquine 32 µM, DB1127 6.1 µM, [14], [16]). The pronounced impact of BTZ on the structural integrity of the germinal layer of *E. multilocularis* metacestodes was confirmed by electron microscopical assessment. At a concentration of 0.05 µM the ultrastructure of microtriches was severely affected and clear alterations pointing towards cell death could be observed within the germinal layer. Even though a minor morphological effect on stem cells was observed by electron microscopy, BTZ is expected to be able to efficiently inhibit the growth and survival of germinal layer cells as they are actively metabolizing cells. This is crucial for a new treatment option against AE, since stem cells are the central target to prevent recurrence after drug treatment [32], [33].

BTZ had been developed as a proteasome-inhibitor for the treatment of multiple myeloma and mantle cell lymphoma [20], [21]. In protozoan parasites such as *Plasmodium* spp. [34]–[40], *Leishmania* spp. [41]–[45], *Toxoplasma gondii* [46], *Trypanosoma cruzi* [47], [48] and *T. brucei* [49], [50] the proteasome has been long investigated as a potential drug target. In addition, the anti-malarial activity of BTZ and one of its derivatives was assessed earlier [51]. In helminths, however, the proteasome largely received attention within the nematode species *Caenorhabditis elegans* regarding developmental studies and in *Schistosoma mansoni* regarding studies on development and stress response [52]. As a drug target BTZ has not been experimentally addressed so far. However, it is worth mentioning that within the framework of the recently completed *Echinococcus* and *Taenia* genome sequencing project, the proteasome has been implicated as a potential drug target [7].

The proteasome of eukaryotes is built of a core particle (20S) and one or two regulatory particles (19S) that together form the 26S proteasome [53]. The 26S proteasome degrades proteins that are conjugated to ubiquitin in an ATP-dependent manner. In addition the 20S proteasome degrades unfolded, damaged, mutated and short-lived proteins also in an ubiquitin- and ATP-independent manner [54]. Thereby the proteasome takes over a critical role in regulating cell survival and cell cycle control [55]. In cancer cells, the inhibition of the proteasome leads to accumulation of proteasome-regulated proteins such as cell cycle-regulatory proteins, cyclin-dependent kinases, tumor suppressors and transcription factors [55], [56]. One of these is NFKB that is attributed a crucial role in the activity of BTZ [55], [56], but in cestodes the NFKB pathway does not exist [7]. Upon treatment of cancer cells with BTZ, reduced degradation of proteins of cell cycle control leads to apoptosis [55], [56]. Induction of apoptosis has been previously postulated as a strategy to target cestode metacestodes [7] and certain hallmarks of apoptosis have been observed in protoscoleces of *E. multilocularis* [57], [58]. Overall, inhibition of the proteasome causes multiple effects, as this leads to functional impairment of multiple proteasome-regulated proteins, especially in actively dividing cells such as the *E. multilocularis* stem cells. This makes the proteasome a very attractive drug target. Therefore we further assessed the mode of action of BTZ in the cestode *E. multilocularis* and the proteasome as a potential drug target.

Exposure of in vitro-cultured *E. multilocularis* metacestode vesicles to BTZ led to an accumulation of ubiquitinated proteins, as expected for a drug that targets the proteasome. In yeast, BTZ was described to inhibit the chymotryptic activity of the proteasome by binding through its boronic acid residue to the beta 5 subunit of the core particle, PSMB5 [23]. To a lesser extent BTZ can bind also to the beta 1 subunit PSMB1, but this was not further followed within this study [29]. To confirm that BTZ also inhibited chymotrypsin-like activity of the *E. multilocularis* proteasome, specific substrate cleavage was performed in solution, and the calculated IC_50_ of BTZ was 0.76 µM, which largely corresponds to the EC_50_ measured for metacestodes (0.6 µM), and which is also in the range of the IC_50_ies of BTZ in cancer cells (0.1 µM; [55]). Since BTZ inhibited the substrate cleavage activity only to a certain degree, Halt protease inhibitor mix was added. The effects of Halt protease inhibitor revealed that other, non-BTZ sensitive proteases were cleaving the substrate in use. It can, however, not be completely excluded that Halt also affects the chymotrypsin-like activity of the proteasome, as it has been shown by Yabe et al that one component (Aprotinin) has inhibiting activity on all three active subunits of the proteasome [30]. Nevertheless, we included Halt protease inhibitor, because only by this a stable DMSO control could be achieved. Thus, in-gel assays were performed using the same substrate, which revealed that only one band of chymotryptic activity was visible that could be partially inhibited by BTZ.

Since BTZ inhibits the chymotryptic activity of the proteasome [23], an ortholog to the beta 5 subunit was searched for in the *E. multilocularis* genome data base, and one protein (EmPSMB5) was identified. Sequence analysis showed that the amino acids that are essential for proteolytic function [22] are conserved in EmPSMB5 (Figure 4). It has to be highlighted that apart from its main target PSMB5, BTZ can inhibit also other targets, such as for example chymotrypsin (as shown in the control of Figure 7B), cathepsins, chymase, and others, because BTZ is not a completely specific inhibitor [59]. Western blot analysis of the chymotryptic active band that could be inhibited by BTZ showed that EmPSMB5 had the same apparent molecular mass. Taken together, the proteasome of *E. multilocularis* represents a valuable drug target for inhibitors that have little or no activity against the proteasome of the host.

In an attempt to translate these in vitro findings into in vivo efficacy, experimentally infected mice were treated with BTZ or a combination of ABZ and BTZ, and the treatment effects were compared to a standard ABZ treatment, or no treatment at all. Morphological assessments showed that metacestodes from BTZ-treated animals were encapsulated by connective tissue, which limited proliferation. The degree of encapsulation was even more pronounced in ABZ and ABZ/BTZ-treated mice. The reduction in parasite weight, although evident in BTZ-treated mice compared to the non-treated control, was not statistically significant. In contrast the standard treatment with ABZ resulted in a significant reduction of parasite weight compared to the control. One of the reasons for the only moderate effectiveness of BTZ against AE might be the model of secondary infection, which results in parasite proliferation mainly in the abdomen. Upon i.v. application of BTZ high drug levels could be detected in the liver, but not in the abdomen [55] and BTZ was shown to be limitedly active against solid tumors, as in contrast to hematologic tumors [60]. Thus, in future in vivo studies in mice, primary (egg-) infection should be performed that will result in parasite growth mainly in the liver, and which would be more representative for the natural situation and thus would reflect a more realistic scenario.

The in vivo study confirmed previously described adverse effects of BTZ in mice such as diarrhea, fatigue and neuropathy [61]. Although the initial treatment regime was based on previously published studies [62]–[65], it had to be adjusted to a lower dosage of BTZ than recommended due to the increased occurrence of adverse side-effects that occurred upon prolonged duration of treatment. However, it has also been shown that even though BTZ induces neuropathy in 31–64% of the patients, there is full neurological recovery in 86% of the cases, because BTZ binds reversibly to the proteasome and after treatment all nucleated, surviving cells can resynthesize new proteasomes [60]. For other cytostatic drugs the recovery rate is usually lower (64–71%) [66].

In conclusion, we here demonstrate for the first time that a proteasome inhibitor, BTZ, exhibits an excellent in vitro activity against the cestode *E. multilocularis*. This study identifies the proteasome as a highly interesting and valuable drug target in cestodes. The divergence of the *E. multilocularis* proteasome from the host cell proteasome implies that more selective inhibition of the cestode proteasome should be feasible in future. The in vivo activity of BTZ in the secondary mouse model for alveolar echinococcosis did not provide satisfying results. Thus, further adjustment of treatment regimens will be pursued. In parallel, new generation proteasome inhibitors that exhibit better bioavailability, higher selectivity and lower host toxicity [60] provide novel starting points for the development of a treatment option for AE and other cestode infections that is based on inhibiting the functional activity of the proteasome.

## Supporting Information

Table S1 **Complete results of the FDA library screening by PGI assay.** The complete list of all 426 drugs tested in singlets at 20 µM is given together with the percentage activity of each drug in relation to the positive control DB1127 at 20 µM.
(XLSX)
